# Some Refinements and Generalizations of I. Schur Type Inequalities

**DOI:** 10.1155/2014/709358

**Published:** 2014-03-16

**Authors:** Xian-Ming Gu, Ting-Zhu Huang, Wei-Ru Xu, Hou-Biao Li, Liang Li, Xi-Le Zhao

**Affiliations:** ^1^School of Mathematical Sciences, University of Electronic Science and Technology of China, Chengdu 611731, China; ^2^School of Science, North University of China, Taiyuan 030051, China

## Abstract

Recently, extensive researches on estimating the value of *e* have been studied. In this paper, the structural characteristics of I. Schur type inequalities are exploited to generalize the corresponding inequalities by variable parameter techniques. Some novel upper and lower bounds for the I. Schur inequality have also been obtained and the upper bounds may be obtained with the help of *Maple* and automated proving package (*Bottema*). Numerical examples are employed to demonstrate the reliability of the approximation of these new upper and lower bounds, which improve some known results in the recent literature.

## 1. Introduction

It is well known that *x*_*n*_ = (1 + (1/*n*))^*n*^ and *y*_*n*_ = (1 + (1/*n*))^*n*+1^ are, respectively, monotone increasing and monotone decreasing, and both of them converge to the constant *e*. In fact, extensive researches for the estimated value of *e* have been studied [1–4], and the methods for estimating the value of *e* are of benefit to the improvements of the Hardy inequality, Carleman inequality, Gamma function inequality, and so forth [5–13], which is an essential motivation for this work. Klambauer and Schur have reached the following conclusion.

**Lemma 1 (see [14])**

Both *s*_*n*_ = (1 + (1/*n*))^*n*+*α*^ and *t*_*n*_ = (1 + (1/*n*))^*n*+1^(1 + (*α*/*n*)) are monotone decreasing sequences if and only if *α* ≥ (1/2).

In fact, it is not hard to prove that

(1) $$\begin{array}{c} e<{\left(1+\frac{1}{n}\right)}^{n+(1/2)}=\sqrt{1+\frac{1}{n}}{\left(1+\frac{1}{n}\right)}^{n}, \end{array}$$

which has been proved by different ways; refer to [14–17].

Besides, Fischer and Qi had further studied this issue (see [18–20]) and they demonstrated that *x*_*n*_ is a monotone increasing sequence if and only if

(2) $$\begin{array}{c} \alpha \leq \frac{2\ln3-3\ln2}{2\ln2-\ln3}=0.409\ldots . \end{array}$$

Moreover, Alzer and Qi have obtained the necessary and sufficient conditions for the monotonicity of generalized types of *b*_*n*_ = (1 + (*α*/*n*))^*n*+*β*^ and *F*_*α*,*β*_(*x*) = (1 + (*α*/*x*))^*x*+*β*^; see [21, 22] for details.

Recently, I. Schur has obtained the so-called I. Schur inequality as follows:

(3) $$\begin{array}{c} p_{n}<e<q_{n}, \end{array}$$

where *p*_*n*_ = *x*_*n*_(1 + (1/(2*n* + 1))) and *q*_*n*_ = *x*_*n*_(1 + (1/2*n*)).

It can also solve the problem proposed by Klambauer in [15]: “Is the *e* contained in a quarter of the interval of (1 + (1/*n*))^*n*^ < *e* < (1 + (1/*n*))^*n*+1^?” Therefore, here *e* is included in the interval length of

(4) E=(1+12n)−(1+12n+1)=12n(2n+1)≤16,                   n=1,2,….

In [23], Xu and Yang have obtained a series of improved forms of the I. Schur inequalities, from which there is one conclusion which can be drawn that the necessary and sufficient condition for (1 + (1/*n*))^*n*^(1 + (1/*an*)) < *e* < (1 + (1/*n*))^*n*^(1 + (1/2*n*)) is *a* > (2/(*e* − 2)).

Many new estimated values on *e* have been obtained when researchers study the improvements of the Carleman inequality; see [24–26] for details. We mainly analyze the structural characteristics of I. Schur type inequalities and find out their upper and lower bounds are closely related to the mean value sequences of *x*_*n*_ and *y*_*n*_. At last, we generalized a series of conclusions on the developments of I. Schur inequality via introducing variable parameters.

The rest of work is organized as follows. In Section 2, upper and lower bounds for a series of the I. Schur type inequalities are improved. Meanwhile, novel I. Schur-like inequalities are obtained by means of the variable parameter method. Some numerical examples are given to show the reliability of the approximation of new upper and lower bounds in Section 3. Finally, the paper closes with conclusions in Section 4.

## 2. Main Results and Proofs

### 2.1. Improvements Based on Computation of Mean Value

In this section, we consider the arithmetic mean value *A*_*n*_, geometric mean value *G*_*n*_, logarithmic mean value *L*_*n*_, harmonic mean value *H*_*n*_, and antilogarithmic mean *Ω*_*n*_ of *x*_*n*_ and *y*_*n*_, respectively; they are

(5) An=xn+yn2=(1+(1/n))n+(1+(1/n))n+12 =xn(1+12n),Gn=(xnyn)1/2=[(1+1n)n(1+1n)n+1]1/2 =xn(1+1n)1/2,Hn=2xnynxn+yn=xn(1+12n+1),Ln=yn−xnln⁡yn−ln⁡xn =[(1+(1/n))n+1−(1+(1/n))nln⁡⁡(1+(1/n))n+1−ln⁡⁡(1+(1/n))n] =xnnln⁡(1+(1/n)),Ωn=xnyn(ln⁡yn−ln⁡xn)yn−xn=xn(1+1n)ln⁡xn =xn(n+1)ln⁡⁡(1+1n),

where *H*_*n*_ is a monotone increasing sequence, see [14] for a discussion of this issue, which means that the left-hand side of I. Schur inequality is valid. A lower bound for I. Schur inequality can be obtained via the monotone convergence theorem [27, pp. 87-88]. Moreover, it has been confirmed that *A*_*n*_ and *Ω*_*n*_ are monotone decreasing in [28]: both *A*_*n*_ = *x*_*n*_(1 + (1/2*n*)) and *Ω*_*n*_ = *x*_*n*_(*n* + 1)ln⁡(1 + (1/*n*)) are monotone decreasing sequences, and we obtained two inequalities as follows:

(6) e<xn(1+12n),  e<xn(n+1)ln⁡(1+1n),                  n=1,2,….

The first inequality has verified the rationality of the right-hand side of I. Schur inequality, and the second inequality has put forward a method for sharpening the upper bound of I. Schur inequality because of (1 + (1/2*n*)) > (*n* + 1)ln⁡(1 + (1/*n*)), *n* ∈ N*. Besides, the monotonicity of the remaining mean value sequences *G*_*n*_, *L*_*n*_ should also be checked, and the conclusions can be drawn as follows.

**Proposition 2**

*G*_*n*_ = *x*_*n*_(1 + (1/*n*))^1/2^  (*n* ∈ N*) is a monotone decreasing sequence.

**Remark 3**

(1) We can also continue to construct geometric mean value sequence *Q*_*n*_ = ((1+(1/*n*))^2*n*^(1+(1/*n*))^1/2^)^1/2^ = *x*_*n*_(1 + (1/*n*))^1/4^. By Lemma 1 and (1/4) = 0.25 < ((2ln⁡3 − 3ln⁡2)/(2ln⁡2 − ln⁡3)) = 0.409…, we can obtain that *Q*_*n*_ = *x*_*n*_(1 + (1/*n*))^1/4^ is a monotone decreasing sequence; thus *x*_*n*_(1 + (1/*n*))^1/4^ < *e*.

(2) Consider *m* ≥ 2, *m* ∈ N*, times of geometric mean value operation by combining with Lemma 1 and (1/2^*m*^) < ((2ln⁡3 − 3ln⁡2)/(2ln⁡2 − ln⁡3)) = 0.409…, and we have *Q*_*n*_ = *x*_*n*_(1 + (1/*n*))^1/2^*m*^^ < *e*, which is a monotone increasing sequence for each *m*.

**Theorem 4**

*L*_*n*_ = *x*_*n*_/(*n*ln⁡(1 + (1/*n*))) is a monotone increasing sequence, and the following inequality

(7) $$\begin{array}{c} L_{n}=\frac{x_{n}}{n\ln\left(1+\left(1/n\right)\right)}>e \end{array}$$

holds for *n* ∈ N*.

**Proof**

We consider the monotonicity of *f*(*x*) = (*x*/ln⁡*x*), (1 < *x* < *e*), and

(8) $$\begin{array}{c} f^{\prime }\left(x\right)=\frac{\ln x-1}{{\left(\ln x\right)}^{2}}, \end{array}$$

when *x* ∈ (1, *e*), *f*′(*x*) < 0 and hence *f*(*x*) is monotone decreasing. It is known that *x*_*n*_ = (1 + (1/*n*))^*n*^ is monotone increasing, and *x*_*n*_ = (1 + (1/*n*))^*n*^ ∈ (1, *e*). According to monotonous property of the composite functions, *L*_*n*_ = *x*_*n*_/(*n*ln⁡(1 + (1/*n*))) is a monotone decreasing sequence; thus

(9) $$\begin{array}{c} f\left(x\right)=\frac{x}{\ln x}>f\left(e\right)=\frac{e}{\ln e}=e. \end{array}$$

Take *x* = (1 + (1/*n*))^*n*^ ∈ (1, *e*); then

(10) $$\begin{array}{c} L_{n}=\frac{x_{n}}{n\ln\left(1+\left(1/n\right)\right)}>e. \end{array}$$

Based on this conclusion, it is not hard to note that it is an improvement of upper bound for I. Schur inequality. There is another conclusion shown as follows (see [14]).

**Proposition 5**

The double inequality

(11) $$\begin{array}{c} {\left(1+\frac{1}{n}\right)}^{n+\alpha }\leq e\leq {\left(1+\frac{1}{n}\right)}^{n+\beta } \end{array}$$

for *n* ≥ 1 is valid in the sense that the maximum *α* = (1/ln⁡2) − 1 and minimum *β* = 1/2 in (11) are best possible.

Then we study the necessary and sufficient condition for the validness of the class of (1 + (1/(2*n* + *β*)))*x*_*n*_ ≤ *e* and obtain the following.

**Proposition 6**

The inequality (1 + (1/(2*n* + *β*)))*x*_*n*_ ≤ *e* holds if and only if *β* ≥ (5/6), and *β* = 5/6 is the best constant.

In fact, the proof of this theorem can be introduced via the conclusions in [14] as follows:

(12) $$\begin{array}{c} \frac{6e}{12x+11}<e-{\left(1+\frac{1}{x}\right)}^{x}<\frac{7e}{14x+12}\;\left(x\geq 1\right). \end{array}$$

Use the left-hand side of (12) and change *x* into *n*, *n* ∈ N*; then

(13) $$\begin{array}{c} {\left(1+\frac{1}{n}\right)}^{n}<e-\frac{6e}{12n+11}=\frac{12n+5}{12n+11}e,\;n\in N^{*} \end{array}$$

and we can also obtain (1 + (1/(2*n* + (5/6))))*x*_*n*_ ≤ *e*. The conclusion on the best optimality of *β* can be found in [13]. It needs to be mentioned that the authors have made a mistake when they cited this inequality in [14]; for instance, let *x* = (1/10); then *e* − (1 + (1/*x*))^*x*^ = 1.447300213…, but (7*e*/(14*x* + 12)) = 1.419997970…. It means that the right-hand side of the inequality may not be valid. It is found that they mistake *x* ≥ 1 for *x* > 0 after checking the original paper [8]. In fact, we can prove *e* − (1 + (1/*x*))^*x*^ < (*e*/(2*x* + *α*)), *x* > 0, and the constant *α* = *e*/(*e* − 1) is the best possible.

**Theorem 7**

The sequence

(14) $$\begin{array}{c} S_{n}=\frac{p_{n}+q_{n}}{2}=x_{n}\frac{8n^{2}+8n+1}{8n^{2}+4n} \end{array}$$

is monotone decreasing, and the inequality *x*_*n*_(1 + (4*n* + 1)/(8*n* ^2^ + 4*n*)) > *e* holds for *n* ∈ N*.

**Proof**

We consider *g*(*x*) = ln⁡(8*x* ^2^ + 8*x* + 1) − ln⁡(8*x* ^2^ + 4*x*) + *x*ln⁡(*x* + 1) − *x*ln⁡*x*, *x* > 0; then

(15) g′(x)=16x+88x2+8x+1−16x+48x2+4x +ln⁡(x+1)−ln⁡x−1x+1,g′′(x)=168x2+8x+1−(16x+8)2(8x2+8x+1)2 −168x2+4x+(16x+4)2(8x2+4x)2−1x(x+1)2=(128x6+384x5+488x4+336x3  +125x2+21x+1) ×((8x2+8x+1)2(2x+1)2(x+1)2x2)−1>0,

where *x* > 0. By lim⁡_*x*→*∞*_⁡*g*′(*x*) = 0, we obtain *g*′(*x*) < 0 (*x* > 0). Therefore, *S*_*n*_ is monotone decreasing, and we can straightforwardly prove the desired inequality of the theorem with lim⁡_*n*→*∞*_⁡*S*_*n*_ = *e*.

**Theorem 8**

The sequence

(16) $$\begin{array}{c} T_{n}=\frac{2p_{n}q_{n}}{p_{n}+q_{n}}=x_{n}\frac{8n^{2}+12n+4}{8n^{2}+8n+1} \end{array}$$

is monotone decreasing, and the following inequality

(17) $$\begin{array}{c} x_{n}\frac{8n^{2}+12n+4}{8n^{2}+8n+1}=x_{n}\left(1+\frac{4n+3}{8n^{2}+8n+1}\right)>e \end{array}$$

holds for *n* ∈ N*.

**Proof**

We consider *h*(*x*) = ln⁡(8*x* ^2^ + 12*x* + 4) − ln⁡(8*x* ^2^ + 8*x* + 1) + *x*ln⁡(*x* + 1) − *x*ln⁡*x*, *x* ≥ 1, then *h*′(*x*) = ((16*x* + 12)/(8*x* ^2^ + 12*x* + 4)) − ((16*x* + 8)/(8*x* ^2^ + 8*x* + 1)) + ln⁡(*x* + 1) + (*x*/(*x* + 1)) − ln⁡*x* − 1, and

(18) h′′(x)=168x2+12x+4−(16x+12)2(8x2+12x+4)2−168x2+8x+1 +(16x+8)2(8x2+8x+1)2+2x+1−x(x+1)2−1x=128x4+256x3+152x2+24x−1x(x+1)(8x2+8x+1)2(2x+1)2>0,

where *x* ≥ 1. According to lim⁡_*x*→*∞*_⁡*h*′(*x*) = 0, we obtain *h*′(*x*) < 0 (*x* ≥ 1). Therefore, *T*_*n*_ is monotone decreasing, and we can straightforwardly prove the desired inequality with lim⁡_*n*→*∞*_⁡*T*_*n*_ = *e*.

In fact, the previous theorems can be viewed as the different improvements of original I. Schur inequality. The above improved inequalities follow the motivation of researching mean value sequences of the upper and lower bounds for original inequalities; the authors study the monotonicity of the sequences constructed by the mean value of upper and lower bounds for I. Schur inequality and its relationship with *e*. The arithmetic mean value should be replaced with the “weighted” mean value *W*_*n*_ = (*x*_*n*_ + *λy*_*n*_)/(1 + *λ*) = [1 + *λ*/(*λ* + 1)*n*]*x*_*n*_, *λ* ∈ ℝ; then we have the following.

**Theorem 9**

For $\lambda \in [\left(-1-\sqrt{3}\right)/4,\left(-1+\sqrt{3}\right)/4]$ and *n* ∈ N*, the sequence *W*_*n*_ is monotone decreasing, and the inequality *x*_*n*_[1 + *λ*/(*λ* + 1)*n*] < *e* holds.

**Proof**

We consider *d*(*x*) = ln⁡(*x* + *λx* + *λ*) − ln⁡(*x* + *λx*) + *x*ln⁡(*x* + 1) − *x*ln⁡*x*  (*x* > 0); then

(19) d′(x)=1+λx+λx+λ−1+λx+λx+ln⁡(x+1) +xx+1−ln⁡x−1,

(20) d′′(x)=−(1+λ)2(x+λx+λ)2+(1+λ)2(x+λx)2+2x+1 −x(x+1)2−1x=(2λx2+3λ2x2+3λ2x  +x3λ2−x3+λ2+2λx) ×((x+1)2x2(x+λx+λ)2)−1.

Now rewrite numerator of (20) and denote *I*_*x*_(*λ*) = (3*x* ^2^ + 3*x* + *x* ^3^ + 1)*λ* ^2^ + (2*x* + 2*x* ^2^)*λ* − *x* ^3^. This is a quadratic function of *λ*, and its discriminant is

(21) $$\begin{array}{c} \Delta ={\left(2x+2x^{2}\right)}^{2}+4\left(3x^{2}+3x+x^{3}+1\right)x^{3}>0. \end{array}$$

We note that two roots of *I*_*x*_(*λ*) are

(22) λ1=−(1+1+x2+x)x(x+1)2,λ2=(−1+1+x2+x)x(x+1)2.

It is not hard to prove that ${\left\{-\left((1+\sqrt{1+x^{2}+x}\text{)}x/{\left(x+1\right)}^{2}\right)\right\}}_{x=1}^{\infty }$ is strictly monotone decreasing, and

(23) $$\begin{array}{c} -1<-\frac{\left(1+\sqrt{1+x^{2}+x}\right)x}{{\left(x+1\right)}^{2}}\leq -\frac{1+\sqrt{3}}{4}=-0.6830127\ldots , \end{array}$$

and ${\left\{(-1+\sqrt{1+x^{2}+x})x/{\left(x+1\right)}^{2}\right\}}_{x=1}^{\infty }$ is strictly monotone increasing; we also have

(24) $$\begin{array}{c} \frac{-1+\sqrt{3}}{4}=0.1830127\ldots \;\leq \frac{\left(-1+\sqrt{1+x^{2}+x}\right)x}{{\left(x+1\right)}^{2}}<1. \end{array}$$

Hence, the inequality *I*_*x*_(*λ*) ≤ 0 always holds for *x* ≥ 1 and $\lambda \in [\left(-1-\sqrt{3}\right)/4,\left(-1+\sqrt{3}\right)/4]$. By using lim⁡_*x*→*∞*_⁡*d*′(*x*) = 0, thus *d*′(*x*) ≥ 0. It shows that sequence *W*_*n*_ = [1 + *λ*/(*λ* + 1)*n*]*x*_*n*_ is monotone increasing under this condition, and the inequality in this theorem can be proved by using lim⁡_*n*→*∞*_⁡*W*_*n*_ = *e*.

### 2.2. Further Discussions Based on Introducing the Parameters

Here we study the monotonicity of two new sequences and their relationships with *e* by introducing real parameter *λ*. Two sequences are, respectively, *i*_*n*_ = (1 + (*λ*/*n*))^*n*^ and *j*_*n*_ = (1 + (*λ*/*n*))^*n*+1^, where *λ* > 0, and it is easy to obtain lim⁡_*n*→*∞*_ *i*_*n*_ = lim⁡_*n*→*∞*_ *j*_*n*_ = *e* ^*λ*^. Next, we consider their arithmetic mean value sequence, geometric mean value sequence, and harmonic mean value sequence, respectively; consider

(25) $$\begin{array}{c} A_{1}\left(n,\lambda \right)=\frac{i_{n}+j_{n}}{2}=\left(1+\frac{\lambda }{2n}\right){\left(1+\frac{\lambda }{n}\right)}^{n},\\ G_{1}\left(n,\lambda \right)={\left(i_{n}j_{n}\right)}^{1/2}={\left(1+\frac{\lambda }{n}\right)}^{1/2}{\left(1+\frac{\lambda }{n}\right)}^{n},\\ H_{1}\left(n,\lambda \right)=\frac{2i_{n}j_{n}}{i_{n}+j_{n}}=\left(1+\frac{\lambda }{2n+\lambda }\right){\left(1+\frac{\lambda }{n}\right)}^{n}. \end{array}$$

**Theorem 10**

For 0 < *λ* ≤ 1 and *n* ∈ N*, the sequences *A*_1_(*n*, *λ*) = (1 + (*λ*/2*n*))(1 + (*λ*/*n*))^*n*^ are monotone decreasing, and the inequality *e* ^*λ*^ < (1 + (*λ*/2*n*))(1 + (*λ*/*n*))^*n*^ holds.

**Proof**

We consider *M*(*x*) = *x*ln⁡(*x* + *λ*) − *x*ln⁡*x* + ln⁡(2*x* + *λ*) − ln⁡(2*x*), *x* > 0; then

(26) M′(x)=ln⁡⁡(x+λ)+xx+λ−ln⁡x−1+22x+λ−1x,M′′(x)=21x+λ−x(x+λ)2−1x−4(2x+λ)2+1x2=−(λ(4x3λ+4x2λ2+xλ3−4x3−9x2λ    −6xλ2−λ3))×(x2(x+λ)2(2x+λ)2)−1.

Denote the numerator of *M*′′(*x*) in (26) as

(27) N(x)≔−λ(4x3λ+4x2λ2+xλ3−4x3−9x2λ    −6xλ2−λ3)≔λY(x),

where *Y*(*x*): = 4*x* ^3^ − 4*x* ^3^ *λ* + 9*x* ^2^ *λ* − 4*x* ^2^ *λ* ^2^ + 6*xλ* ^2^ − *xλ* ^3^ + *λ* ^3^. Take ∀ *p* ≥ 0; then *λ* : = 1/(*p* + 1) ∈ (0,1] and

(28) Y(x)∶=4x3−4x3p+1+9x2p+1−4x2(p+1)2+6x(p+1)2 −x(p+1)3+1(p+1)3=(4x3p3+8x3p2+4x3p+9x2p2  +14x2p+5x2+6xp+5x+1)×((p+1)3)−1.

It is found that if 0 < *λ* ≤ 1 and *x* > 0, then *Y*(*x*) ≥ 0. Furthermore, it implies that

(29) $$\begin{array}{c} N\left(x\right)=\lambda Y\left(x\right)\geq 0; \end{array}$$

then *M*′(*x*) is monotone increasing, and lim⁡_*x*→*∞*_⁡*M*′(*x*) = 0 has been verified, such that *M*′(*x*) < 0, and *A*_1_(*n*, *λ*) = (1 + (*λ*/2*n*))(1 + (*λ*/*n*))^*n*^ are monotone decreasing; hence *e* ^*λ*^ < (1 + (*λ*/2*n*))(1 + (*λ*/*n*))^*n*^ can be proved because of lim⁡_*n*→*∞*_⁡*A*_1_(*n*, *λ*) = *e* ^*λ*^.

**Theorem 11**

For 0 < *λ* ≤ 1 and *n* ∈ N*, the sequences *G*_1_(*n*, *λ*) = (1 + (*λ*/*n*))^1/2^(1 + (*λ*/*n*))^*n*^ are monotone decreasing, and the inequality (1 + (*λ*/*n*))^1/2^(1 + (*λ*/*n*))^*n*^ > *e* ^*λ*^ holds.

**Proof**

We consider *f*_1_ = *x*ln⁡(1 + (*λ*/*x*)) + (1/2)ln⁡(1 + (*λ*/*x*)), *x* ≥ 1; then

(30) $$\begin{array}{c} f_{1}^{\prime }\left(x\right)=\ln\left(1+\frac{\lambda }{x}\right)-\frac{\lambda }{x\left(1+\left(\lambda /x\right)\right)}-\frac{\lambda }{2x^{2}\left(1+\left(\lambda /x\right)\right)}, \end{array}$$

(31) f1′′(x)=−λ2x3(1+(1/x))2+λx3(1+(λ/x)) −λ22x4(1+(λ/x))2=−λ(2λx−2x−λ)2x2(x+λ)2=(−2x+1)λ2+2λx2x2(x+λ)2.

Denote the numerator of (31) as *δ*(*λ*) = (−2*x* + 1)*λ* ^2^ + 2*xλ*, which is a quadratic function of *λ*. And its discriminant is Δ_1_ = 4*x* ^2^ > 0. Then two roots of *δ*(*λ*) are

(32) $$\begin{array}{c} \begin{array}{cc} & \lambda_{1}=0,\;\;\lambda_{2}=\frac{2x}{2x-1}. \end{array} \end{array}$$

We note that {2*x*/(2*x* − 1)}_*x*=1_^*∞*^ is strict monotone decreasing and 1 < (2*x*/(2*x* − 1)) ≤ 2, *x* ≥ 1. According to the characteristic of parabola curve, *δ*(*λ*) = (−2*x* + 1)*λ* ^2^ + 2*xλ* > 0 always holds for 0 < *λ* ≤ 1 and *x* ≥ 1; then from lim⁡_*x*→*∞*_⁡*f*_1_′(*x*) = 0, *f*_1_′(*x*) ≤ 0. It shows that the sequences *G*_1_(*n*, *λ*) are monotone decreasing.

**Theorem 12**

If *λ* ≥ 1, then *H*_1_(*n*, *λ*) = (2*i*_*n*_ *j*_*n*_)/(*i*_*n*_ + *j*_*n*_) = (1 + *λ*/(2*n* + *λ*))(1 + (*λ*/*n*))^*n*^ are monotone increasing, and the following inequality

(33) $$\begin{array}{c} \left(1+\frac{\lambda }{2n+\lambda }\right){\left(1+\frac{\lambda }{n}\right)}^{n}<e^{\lambda } \end{array}$$

holds for *n* ∈ N*.

**Proof**

We consider *φ*(*x*) = ln⁡(2*x* + 2*λ*) − ln⁡(2*x* + *λ*) + *x*ln⁡(*x* + *λ*) − *x*ln⁡*x*, *x* > 0; then

(34) $$\begin{array}{c} \varphi^{\prime }\left(x\right)=\frac{2}{2x+2\lambda }-\frac{2}{2x+\lambda }+\ln\left(x+\lambda \right)+\frac{x}{x+\lambda }-\ln x-1, \end{array}$$

(35) φ′′(x)=−4(2x+2λ)2+4(2x+λ)2+2x+λ−x(x+λ)2 −1x=−(λ(−4x2−3λx+4λx2+4xλ2+λ3)) ×((x+λ)2(2x+λ)2x)−1.

Denote numerator of (35) as

(36) $$\begin{array}{c} \gamma \left(x\right)=-\lambda \left(-4x^{2}-3\lambda x+4\lambda x^{2}+4x\lambda^{2}+\lambda^{3}\right)≔-\lambda Z\left(x\right), \end{array}$$

where *Z*(*x*)∶ = −4*x* ^2^ − 3*λx* + 4*λx* ^2^ + 4*xλ* ^2^ + *λ* ^3^. Since *τ* + 1∶ = *λ* ≥ 1, that is, *τ* ≥ 0, we have

(37) Z(x)∶=−4x2−3x(τ+1)+4x2(τ+1) +4x(τ+1)2+(τ+1)3=4x2τ+(τ+1)(4τ+1)x+(τ+1)3≥0.              (x>0,  τ≥0)

It implies that the inequality *γ*(*x*) = −*λZ*(*x*) ≤ 0 always holds for *x* > 0, *λ* ≥ 1. Then *φ*′(*x*) is monotone decreasing, and according to lim⁡_*x*→*∞*_⁡*φ*′(*x*) = 0, we have *φ*′(*x*) > 0. Hence, *H*_1_(*n*, *λ*) = (1 + *λ*/(2*n* + *λ*))(1 + (*λ*/*n*))^*n*^ are monotone increasing sequences, and we prove that

(38) $$\begin{array}{c} e^{\lambda }>\left(1+\frac{\lambda }{2n+\lambda }\right){\left(1+\frac{\lambda }{n}\right)}^{n}. \end{array}$$

A remaining issue of Theorem 12 can be described as follows: when *λ* < 1, are the *H*_1_(*n*, *λ*) monotone increasing or monotone decreasing? With the help of the* Bottema* (available at the site of http://old.irgoc.org/Soft/ShowSoft.asp?SoftID=15, and the practical implementing* Maple* codes also are available by the authors' email) (developed by Yang and Xia and based on the* Maple* programme; see [29] and references therein), we can find that the inequality *γ*(*x*) ≥ 0 holds for 0 < *λ* ≤ (4/5), *x* ≥ 1. Moreover, it can be also shown in Algorithm 1.

Then we can conclude that *H*_1_(*n*, *λ*), *n* = 1,2,…, is monotone decreasing under the previous discussion in the proof of the Theorem 12. The further conclusions need to be studied and proven by analytical methods in future work.

## 3. Numerical Examples

In this section, we will display some new upper and lower bounds of the I. Schur inequality by using the Matlab 2011b in a personal computer. We give some figures to show their variation trend; the symbols *L*_0_–*L*_4_ here mean the corresponding real continuous function for *L*_0_ = *x*_*n*_(1 + (1/2*n*)), *L*_1_ = *x*_*n*_(*n* + 1)ln⁡(1 + (1/*n*)), *L*_2_ = (*x*_*n*_/(*n*ln⁡(1 + (1/*n*)))), *L*_3_ = *x*_*n*_(1 + ((4*n* + 1)/(8*n* ^2^ + 4*n*))), and *L*_4_ = *x*_*n*_(1 + ((4*n* + 3)/(8*n* ^2^ + 8*n* + 1))), respectively.

**Figure 1:**
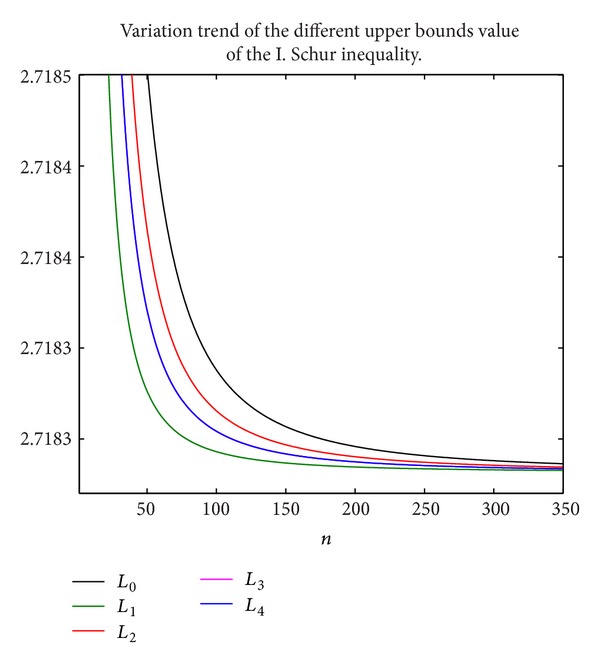
The variation trend of the different upper bounds value of the I. Schur inequality.

Form Figure 1, we can note that the original upper bound *L*_0_ of the I. Schur inequality is not better than the other novel upper bounds *L*_1_–*L*_4_; the *L*_1_ is the best upper bound among five upper bounds. Moreover, we note that the variation curves of *L*_3_, *L*_4_ are greatly similar; they are both the other alternatives. This figure also shows that the improvement of the upper bound of the I. Schur inequality is beneficial.

**Figure 2:**
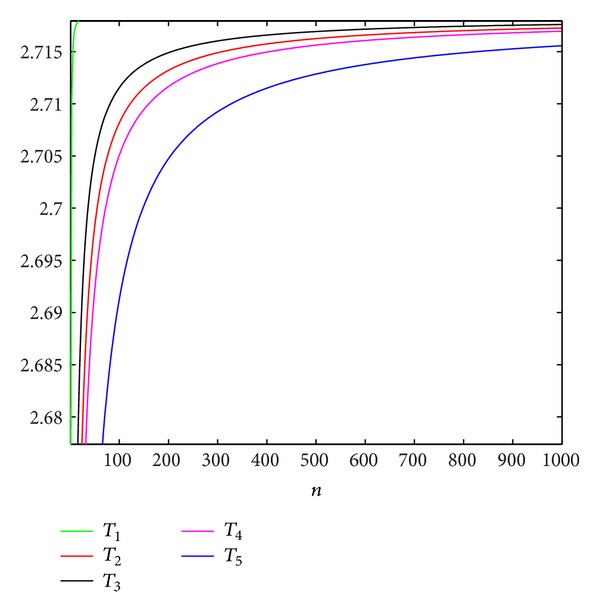
The variation trend of the different lower bounds value of the I. Schur inequality.

Firstly, we define the corresponding real continuous function for *T*_1_ = *x*_*n*_(1 + (1/(2*n* + 1))),   *T*_2_ = *x*_*n*_(1 + (1/*n*))^1/8^, *T*_3_ = *x*_*n*_(1 + (1/*n*))^1/4^,   *T*_4_ = *x*_*n*_(1 + ((1/100)/(1 + (1/100))*n*)), and *T*_5_ = *x*_*n*_(1 + (−1/3)/(1 − (1/3))*n*), respectively. From Figure 2, we can note that the original lower bound *T*_1_ of the I. Schur inequality is better than the other novel lower bounds *T*_2_–*T*_5_; the *T*_3_ is an alternative among other novel lower bounds. This figure shows that we improve the lower bound of the I. Schur inequality and it is not very successful. But we give some generalized upper and lower bounds for the *e* ^*λ*^, *λ* ∈ (0,1); this work is meaningful in terms of the generalized type of the I. Schur inequality.

From Table 1, we find that *H*_1_(*n*, *λ*) can be viewed as a good lower bound of *e* ^*λ*^, *λ* ≥ 1 in terms of approximating precision, especially for the case with *λ* = 1. It is favorable to note that the reliability of the approximation of *e* ^*λ*^ based on *H*_1_(*N*, *λ*) depends on the closeness between *λ* and 1.

## 4. Conclusions

In the previous sections, we have studied the monotonicity of the sequences and their variants based on various mean values of *x*_*n*_ and *y*_*n*_, such as the arithmetic mean value, geometric mean value, logarithmic mean value, and harmonic mean value. Besides, we have given some extensions and remarks for known results in the recent literature. At the same time, we extend some ideas and conclusions in [26, 30–32]. In conclusion, it is not hard to find that the conclusions of previous generalized sequences with variable parameters are in terms of the I. Schur type inequality via the parameter techniques and are a kind of extensions of I. Schur type inequality.

## Figures and Tables

**Algorithm 1:**
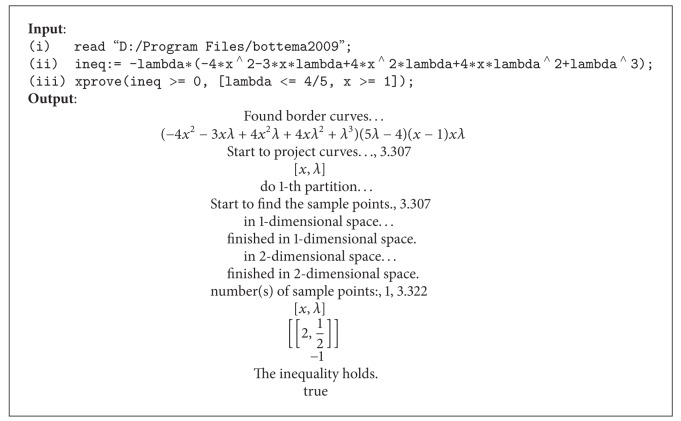


**Table 1:** Using *H*_1_(*N*, *λ*) to approximate the *e* ^*λ*^, when *λ* = 1,3/2,2, 3,4 with different *n*.

*n*	*H*_1_(*n*, 1)	*H*_1_(*n*, 2)	*H*_1_(*n*, 3)	*H*_1_(*n*, 4)	*H*_1_(*n*, 3/2)
	*e* ≈ 2.71828	*e* ^2^ ≈ 7.3891	*e* ^3^ ≈ 20.0855	*e* ^4^ ≈ 54.5982	*e* ^3/2^ ≈ 4.481689
1000	2.71828	7.3817	20.0255	54.2724	4.480010
3000	2.71828	7.3866	20.0655	54.4892	4.481129
5000	2.71828	7.3876	20.0735	54.5327	4.481353
7000	2.71828	7.3880	20.0769	54.5514	4.481449
9000	2.71828	7.3882	20.0788	54.5618	4.481502
11000	2.71828	7.3884	20.0801	54.5684	4.481536
13000	2.71828	7.3885	20.0809	54.5730	4.481560
15000	2.71828	7.3886	20.0815	54.5763	4.481577
